# Promoting clinical reasoning in undergraduate Family Medicine curricula through concept mapping: a qualitative approach

**DOI:** 10.1007/s10459-024-10353-z

**Published:** 2024-06-24

**Authors:** Marta Fonseca, Pedro Marvão, Patrícia Rosado-Pinto, António Rendas, Bruno Heleno

**Affiliations:** 1 Comprehensive Health Research Centre, NOVA Medical School, Lisbon, Portugal; 2 NOVA Medical School, Campo dos Mártires da Pátria, 130, 1169-056 Lisbon, Portugal

**Keywords:** Concept map, Clinical reasoning, Undergraduate medical education, Multimorbidity, Family Medicine

## Abstract

Clinical reasoning is a crucial skill for physicians, enabling them to bridge theoretical knowledge with practical application. The gap between basic sciences and clinical practice persists as a challenge, with traditional teaching methods yet to effectively bridge it. Concept maps (CMs), visual tools for organizing and connecting knowledge, hold promise for enhancing clinical reasoning in the undergraduate medical curriculum. However, further research is required to ascertain if CMs facilitate clinical reasoning development in medical students transitioning from basic sciences to clinical practice. This study aims to delineate how CMs can facilitate clinical reasoning in patients with multimorbidity within undergraduate Family Medicine curricula, as perceived by students and tutors, and to understand the implementation process and resources required. This exploratory qualitative study formed a part of an action research project. While introducing an educational intervention to 5th-year medical students, we conducted a qualitative evaluation. Subsequently, semi-structured group interviews were conducted with students, and a focus group was conducted with tutors. Three main educational impacts were identified: integration of clinical information, support for patient management and care plan, and collaborative learning. Key aspects for successful CM implementation included clear instructions for map construction, using user-friendly software, allocating sufficient time for the task, encouraging group discussion of CMs, and incorporating tutor feedback. CMs are pedagogical tools that facilitate clinical information integration and support management and treatment plans, helping students better understand multimorbidity patients and promoting some components of clinical reasoning in undergraduate medical education.

**Supplementary Information:**

The online version contains supplementary material available at 10.1007/s10459-024-10353-z.

## Introduction

Undergraduate medical education serves as the cornerstone upon which future physicians construct their knowledge and acquire basic clinical skills. It encompasses a dual approach that incorporates theoretical foundations and practical applications. The former establishes the background for comprehending the intricate functioning of the human body, disease mechanisms, and therapeutic modalities. During the clinical experience process, students embark on a journey into the real-world setting of patient care, where they apply their theoretical knowledge under the guidance of experienced tutors. However, the persistent gap between theory and practice calls for the implementation of pedagogical approaches that encourage reflection both on clinical practice (Swanwick, 2019) and on the reasoning behind clinical decisions. More than simply acquiring knowledge, cultivating metacognitive strategies—critically analyzing what you do and why you do it—may contribute to enable medical students to bridge this gap and build strong clinical reasoning skills (Clark & Dumas, 2016).

Clinical reasoning, an essential skill of a physician’s competence, is often also referred to as critical thinking, decision-making, problem-solving, clinical judgment, and diagnostic reasoning. Despite their immense importance, there is a lack of clarity regarding the terminology and distinctions between these concepts (Brentnall et al., 2022). For the purpose of this manuscript, clinical reasoning is conceptualized as a broad umbrella term encompassing reasoning skills, reasoning performance, reasoning processes, outcomes of reasoning, contexts of reasoning, and purpose of reasoning (Young et al., 2020). The complex process of clinical reasoning comprises information gathering, hypothesis generation, problem representation, differential diagnosis, leading or working diagnosis, diagnostic justification, and management and treatment (Daniel et al., 2019).

Emphasis on clinical data integration within the reasoning process is deemed beneficial for developing clinical reasoning. Conventional pedagogical methods, such as text-based learning, do not appear to be effective in bridging the gap between basic sciences and clinical knowledge integration (Groves et al., 2003). Therefore, innovative pedagogical approaches are needed to provide learning pathways to clinical reasoning through networks of meaningful understanding (Kinchin & Cabot, 2010).

Concept maps (CMs) emerge as flexible tools that facilitate knowledge integration across a wide spectrum of contexts, purposes, and variations in their application (Fonseca et al., 2023). CMs, first introduced by Novak in 1984, are visual representations of knowledge that connect concepts meaningfully (Novak, 2003). CMs are based on Ausubel’s constructivist theory, which posits that the acquisition of new knowledge relies on existing knowledge, promoting long-term retention (meaningful learning) (Ausubel, 2000). CMs are effective tools for enhancing critical thinking skills in undergraduate medical education. They clarify knowledge, improve critical thinking, and address knowledge gaps, fostering meaningful concept connections. This approach not only promotes knowledge acquisition but also its integration and accurate application, contributing to the development of clinical reasoning skills. CMs improve logical organization and clarify relationships between concepts, essential for students' critical thinking and clinical reasoning (Daley et al., 2016; Fonseca et al., 2023). Additionally, CMs help students summarize clinical cases or plan actions, thus advancing their analytical critical thinking and clinical reasoning competencies (Demeester et al., 2010). As visual tools, CMs allow students to create and complete maps that represent integrated knowledge networks. This process enables instructors to assess the depth of students' reasoning processes. However, the impact of CMs on different stages of medical training and across various disciplines remains uncertain. Specifically, their role in transitioning from the basic sciences to more experiential clinical training, and eventually into real-life clinical practice, is not well-defined (Daley & Torre, 2010). Moreover, the lack of studies with control groups makes it unclear whether improvements in clinical reasoning are directly due to this teaching strategy (Pierce et al., 2020). Furthermore, effectively teaching clinical reasoning to medical students, particularly in the context of multiorgan diseases and multimorbidity, requires the use of complex cognitive processes (National Institute for Health and Care Excellence, 2016) and the integration of knowledge from different curricular courses. CMs seem to allow the development of a more granular construction of an integrated network of learning units, but further research is needed to explore their relevance in connecting basic sciences and clinical practice (Daley & Torre, 2010; Pierce et al., 2020).

**Fig. 1:**
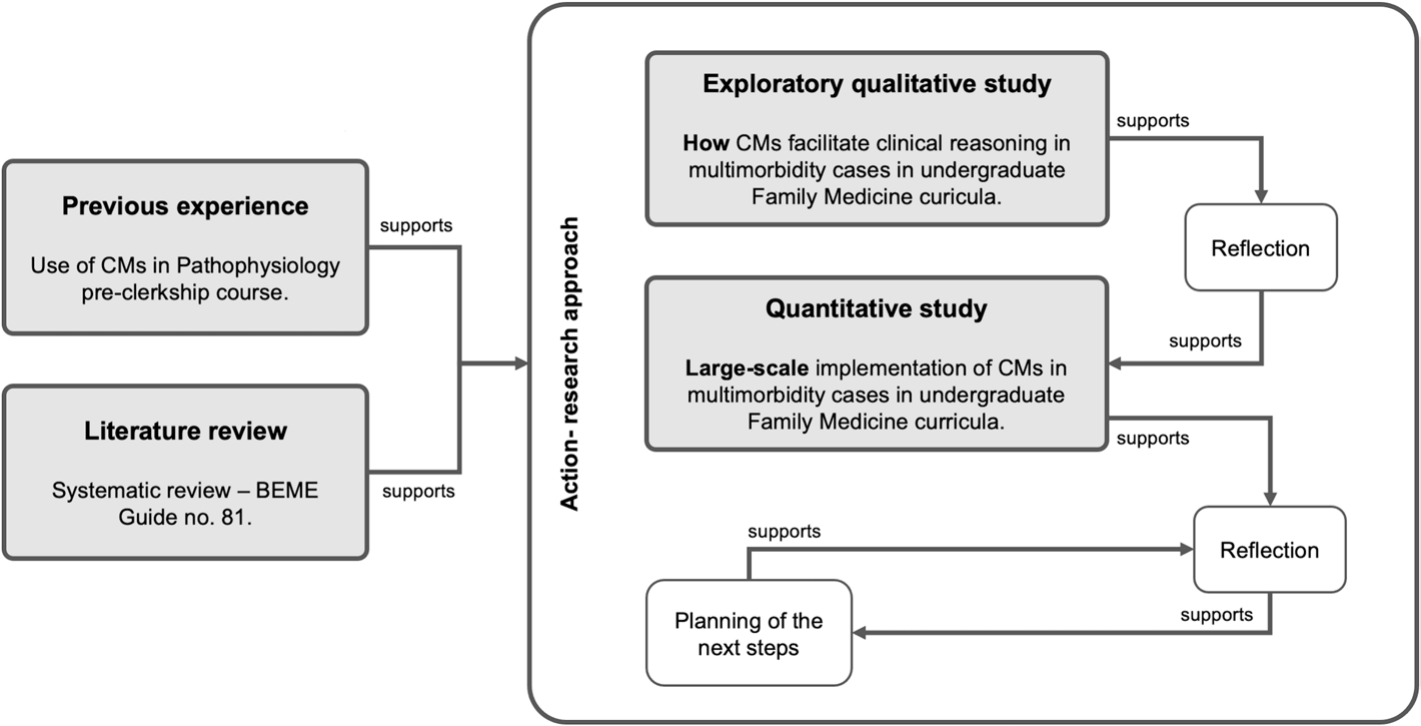
The three components of the research project: a systematic literature review, an exploratory study, and a quantitative large-scale implementation study. CM, concept map

At NOVA Medical School (NMS), CMs have been used since 2002 in the pre-clerkship curricular course of Pathophysiology (Fonseca et al., 2020; Rendas et al., 2006). This experience has enabled the group to develop diverse approaches to using CMs in the basic sciences context. To understand how to implement CMs in the clerkship curriculum, our group is developing a research project to investigate the role of CMs as a tool to facilitate clinical reasoning in medical students (Fig. 1). We began this project with a literature review, which allowed us to better understand how CMs promote critical thinking. This review also indicated that their use varies greatly depending on the local context (Fonseca et al., 2023). Therefore, before proceeding with a large-scale implementation, we conducted a qualitative exploratory study to gain insights into the perspectives of students and tutors in their learning and teaching context. This manuscript describes this qualitative study, which aims to delineate how CMs can facilitate clinical reasoning in patients with multimorbidity within undergraduate Family Medicine curricula, as perceived by students and tutors. In addition, the goal is to gain a better understanding of the implementation process and the resources required.

## Methods

### Qualitative approach

This exploratory qualitative study was part of an action research project based on the interpretivism research paradigm (Bunniss & Kelly, 2010; Swanwick, 2019; Tekin & Kotaman, 2013). While introducing an educational intervention (Meinema et al., 2019) focusing on the use of CMs to enhance clinical reasoning in medical students, we conducted a series of qualitative procedures. As illustrated in Fig. 1, the methodology involved a cyclical process of applying the pedagogical tool, collecting qualitative data (using observational methods, interviews and a focus group), and engaging in reflective analysis by the research team.

### Context

The curriculum at NMS is a 6-year degree and comprises three distinct stages: the first 2 years are pre-clerkship, followed by 3 years of clerkship, and concluding with a transitional year into practice. Within this framework, CMs have traditionally been used in the Pathophysiology course during the pre-clerkship phase (Fonseca et al., 2020; Rendas et al., 2006).

This study aimed to analyze the possibilities of extending the use of CMs within the clerkship Family Medicine course for 5th-year students. In the Family Medicine course, students engage in 1 week of seminars and simulation training, 3 weeks of clinical experience, and a final week of seminars. These seminars, catering to groups of 20 students, focus on discussing clinical cases related to the management of patients with chronic conditions. This study was conducted during the last week of seminars, which occurred outside the clinical environment at the headquarters of the NMS. From the beginning of the academic year, students were randomly assigned to groups of 20, which was maintained throughout the academic year. These groups may include students from diverse backgrounds, such as working students, students with a previous degree, Erasmus^1^ students, scholarship recipients, or student association leaders. Some of these students did not complete the entire initial curriculum of the NMS, such as Erasmus students and some students with a previous degree who had some equivalences in the basic sciences, and therefore had no prior experience in using CMs.

The course was taught by two groups of family physician tutors: academic tutors and clinical tutors. Academic tutors are involved in varying proportions of research, teaching, and clinical activities. On the other hand, clinical tutors primarily undertake clinical work and supervise students in their clerkship, providing the formative assessment.

### Study design

**Fig. 2:**
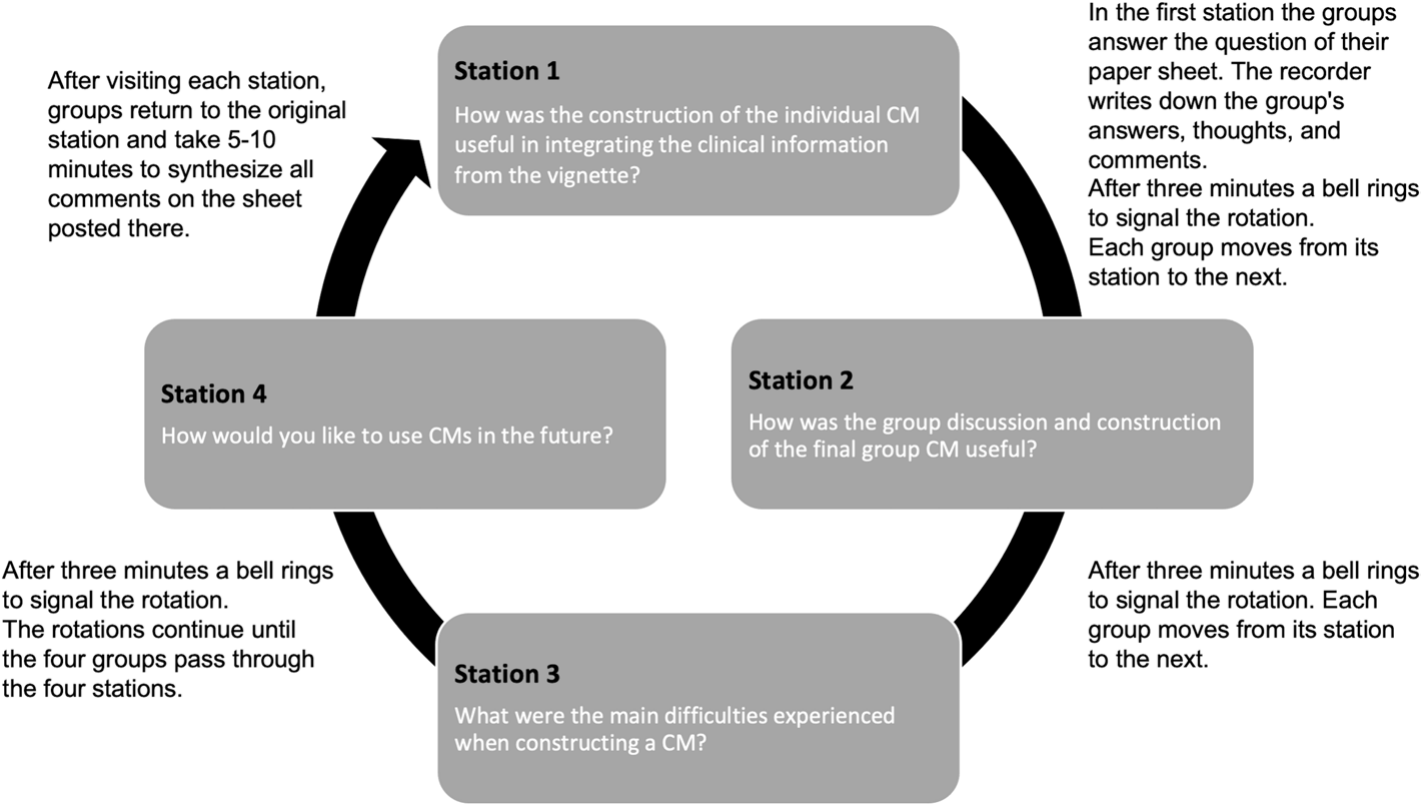
Description of the gallery walk technique (adapted from Francek, 2006). Abbreviations: CM, concept map

The educational intervention sessions were planned as 2-h sessions for 20 students and were conducted between March and April 2022.Three members of the research team served as facilitators. The session started with a 10-min lecture on how to build CMs, based on Pinto and Zeitz (1997) recommendations. This was followed by an individual assignment. Students were given a clinical vignette of a patient with multimorbidity and asked to draw a CM representing the patient on an A3 sheet of paper within a 20-min timeframe. The same clinical vignette was provided to all groups in all intervention sessions (Additional Supporting Information 1 contains the clinical vignette). Afterwards, there was a group activity (4 groups of 5 students per session) where students discussed individual CMs and prepared a final group CM within a 20-min timeframe. After a 10-min break, classroom discussion occurred using the gallery walk technique (Cristancho et al., 2017; Francek, 2006; Helmich et al., 2018). Figure 2 illustrates the dynamics of the gallery walk technique, where the four groups answered four questions about the use of CMs. At the end of the session, each student was asked to write on a post-it note what they most valued from the activity. All the post-it notes were posted on the wall, next to others with similar content, forming a wall of values.

Subsequently, two research team members conducted semi-structured group interviews with students in the first half of July 2022, approximately 3 months after the completion of the intervention sessions, to clarify certain aspects of the intervention. We planned to conduct focus groups with students. However, because of lower than anticipated participant turnout, we adapted our methodology to the situation. We conducted semi-structured group interviews twice to accommodate the available students. This approach, which deviated from our initial plan, was more feasible given the small number of participants in each session. This format allowed for in-depth individual insights within a group setting. It ensured that all participants had the opportunity to share their perspectives on the overall impression of the intervention session, the positive aspects of using CMs, the difficulties encountered, and the potential for future use of CMs (Additional Supporting Information 2 contains the questions for the students).

In late June 2022, we organized a focus group with family physician tutors who were responsible for supervising students during clinical rotations and were not involved with the intervention. This focus group was moderated by two members of our research team. This session began with a brief explanation of the study and the educational intervention implemented and gathered feedback on the advantages and limitations of using CMs (focus group agenda in Additional Supporting Information 2).

### Participants, recruitment, and sampling

Among the 282 students (75.9% female) in the 5th-year of the 2021–2022 academic year, 77 (53 female, 68.8%) attending the Family Medicine course in March and April 2022 were eligible to participate (Table 1). These students were invited to participate in the educational intervention. The first contact was made by the main investigator (MF) through the class representatives. A second contact was made by the course coordinator (BH) during the orientation session. A third contact was made by MF at the start of the intervention session, and informed consent was obtained for participation in this study. Students were informed that the educational intervention session was voluntary, that it would occur in additional face-to-face time at NMS, and that non-participation would not have any influence on their final assessment.

**Table 1:** Overview of the educational intervention sessions, group interviews and focus group

		Duration (minutes)	Eligible participants	Participants, n			
Intervention sessions	Session 1	100	20 (16 F)	10 (6 F)		41	
	Session 2	75	19 (14 F)	2 (2 F)			
	Session 3	100	19 (12 F)	13 (7 F)			
	Session 4	95	19 (11 F)	16 (13 F)			
Group interviews	Group 1	60	14 (12 F)	P01	M	4	7
				P02	F		
				P03	F		
				P04	F		
	Group 2	40	10 (9 F)	P05	F	3	
				P06	F		
				P07	F		

	Duration (minutes)		Participants, n		
Focus group	90	P10	F	clinical tutor (retired)	6
		P11	F	academic tutor	
		P12	F	clinical tutor	
		P13	F	academic tutor	
		P14	F	academic tutor	
		P15	F	clinical tutor	

F, female; M, male; P, participant

During the intervention sessions, key informant students were intentionally identified by the research team. Students of different genders and ages who showed enthusiasm or reservations regarding the use of CMs were actively sought. Semi-structured group interviews were conducted with key informant students to discuss and reflect on the intervention and their perceptions of the usefulness of CMs. Sample sizes were determined based on ongoing data collection and analysis. Student participants were recruited for interviews until thematic saturation was achieved (Saunders et al., 2018).

The focus group was conducted with family physician tutors. These participants were purposefully sampled from a total of 129, based on the number of years as practicing family physicians, their involvement in postgraduate resident training, familiarity with the concept mapping methodology, whether or not they were alumni of NMS, and their availability to participate.

### Data collection and analysis

Data were extracted using direct techniques from:

- Educational intervention sessions: individual and group CMs; the gallery walk exercise record sheets; post-it notes of the wall of values; field notes taken on sheets created specifically for this purpose, as outlined in the session script (MF, PRP, and PM); and feedback from PRP and PM.
- Semi-structured group interviews and the focus group were video recorded. Digital files were only available to MF and one external contractor who transcribed the interviews verbatim. MF validated the transcripts against the recordings, eliminating any identifying information. Field notes were taken by MF and PM.

The intervention sessions, session guides, teaching materials, and informed consent were written in Portuguese, which is the teaching language at our institution.

The research team analyzed the CMs created by the students, focusing on their overall structure, the inclusion of the patient information, and the integration of the diseases and medication. No detailed scoring or further evaluation was conducted.

The qualitative analysis was performed using an inductive approach. Data were lumped together and then coding was applied to all of them by MF, by reading the transcripts, highlighting all the texts considered relevant to the research questions, categorizing the marked texts, and creating new codes. Coding was conducted at least two times for each transcript and discussed with PRP. Through a process of reflection and discussion, MF and BH identified a set of themes drawn from the codes generated across all data sets.

The convergence of information from diverse sources (gallery walk exercise, wall of values, group interviews, and focus group) and the research team’s reflection and discussion enabled us to achieve a more comprehensive understanding of the phenomenon. This triangulation of multiple data sources, along with investigator triangulation, contributed to enhancing the reliability of the study results.

The collected data were analyzed using MAXQDA Analytics Pro 24 (Release 24.1.0, 2023 VERBI GmbH Berlin). The results were reported and structured according to the Standards for Reporting Qualitative Research (O’Brien et al., 2014).

### Research team characteristics, reflexivity, and techniques to enhance trustworthiness

Three members of the research team were directly involved in data acquisition. The lead investigator, MF, holds an MD, specializes in Family Medicine, and has been teaching Pathophysiology using CMs since 2002. This study forms part of her PhD project. MF played multiple roles, including leading educational intervention sessions, conducting group interviews, and moderating the focus group. PM holds a PhD in Biomedical Sciences, is the coordinator of the Education Department at NMS, and has extensive experience in curriculum development and implementation. PM participated in the educational intervention sessions, the group interviews and focus group. PRP, who holds a PhD in Educational Sciences, is a retired university professor and former Pro-Rector for Education at Universidade NOVA de Lisboa. She has over 20 years of experience in directing the Medical Education Office at NMS and has significantly contributed to teacher training and educational consultancy in Europe and Portuguese-speaking countries. PRP participated in the educational intervention sessions. The other two members of the research team were mostly involved in the design of the study and data interpretation. AR holds a PhD in Experimental Pathology, is an emeritus professor of Pathophysiology, and has conducted research on problem-based learning and on CMs in Pathophysiology for medical students. BH, PhD, is a family physician and an assistant professor of Family Medicine. He has been teaching clinical reasoning since 2016 and had little experience with CMs before this study.

This project was conducted with the support of the NMS Education Office.

### Ethical approval

This study protocol was approved by the NOVA Medical School Ethics Committee on 19th January 2022 (No. 214/2021/CEFCM).

## Results

Three main educational impacts were identified: integration of clinical information, support for management and treatment plans, and promotion of collaborative learning. We start this section with a brief description of the participants, general aspects related to the intervention sessions, group interviews, and focus group. Next, we elaborate on the main educational impacts and close with the resources required to implement the intervention.

### Participants and general considerations

Four educational intervention sessions were performed on different groups of students over two days. The total sample consisted of 41 participants (Table 1). Of the 41 students, 28 (68.3%) were female, 6 (14.6%) had a previous degree, and 4 (9.8%) were Erasmus students. The sessions proceeded according to the scripts, except for session 2, in which only two students were recruited, forming a single group. The group discussion of the gallery walk was cut in all sessions because the session ran overtime. The time available for tasks was always used in full, and none of the CMs were finished.

As the sessions progressed, the research team identified a positive response from most participants, reflecting that they were not only engaged but also benefiting from the task. Some students faced some difficulties with the rules for constructing CMs, particularly those who were using this tool for the first time. These students sought support from the research team. Both individual and group CMs were too simple, probably reflecting the lack of time to perform the task. In addition, the fact that no individual feedback was provided may have limited a significant improvement in the group map (some CMs are displayed in Additional Supporting Information 3). The synthesis of the responses to the gallery walk exercise can be found in Additional Supporting Information 4, and the post-it notes exercise with the respective analysis of the students’ responses can be found in Additional Supporting Information 5.

Semi-structured group interviews were conducted in two different days. Four students participated in the first interview, and three students participated in the second interview.

The focus group included six participants. The six tutors were female, one was a retired clinical tutor, two were clinical tutors, and three were academic tutors.

### Main educational impacts

The following main educational impacts are illustrated with the respective quotations in Tables 2, 3, and 4 (more detailed information can be found in Additional Supporting Information 4, 5, and 6).

**Table 2:** Quotations regarding the CMs as a tool to facilitate clinical information integration

Code	Illustrative examples
*CMs facilitate clinical information integration*	
Identification	"(…) the individual map was more for me to familiarize myself with the patient." (P01 in the group interview) "(…) the objective is to identify entities, not only issues that are related to the physician’s agenda, but that are related to the patient's agenda, which identify priorities for manage a plan later." (P13 in focus group)
Organization	"CMs allow for the hierarchical organization of the patient problems." (gallery walk exercise) "CMs promote concepts organization." (post-it note from the wall of values) "(…) organizing ideas is easier with CMs. I make diagrams or tables too." (P04 in the group interview) "(…) in other words, it is as if this CM managed, in a very clear and hierarchical way, to almost decompose some causes that then determine some mechanisms of the disease and the pathogenesis that then leads to these manifestations." (P14 in focus group)
Synthesis	“CMs were useful to do the synthesis of the patient's clinical information with multimorbidity.” (gallery walk exercise) "CMs facilitate information synthesis." (post-it note from the wall of values) "(…) when I have a clinical case to present, I will often create a CM to summarize the patient's information." (P03 in the group interview)
Integration	"I can only make a good problem map to support the therapeutic plan if I can make this CM. If I can't make the CM, this background issue, I can't resolve the foreground issues.” "(…) this map of concepts can, in a very clear and hierarchical way, identify some causes that determine some mechanisms of the disease and the pathogenesis of the manifestations." (P14 in focus group)
Visual Representation	"(…) visually it is much easier." (P05 in the group interview) “It is an individual matter, that is, there are people who identify very much with CMs, I happen to be one of them, but there are others who identify much more with text. (…) while it is very easy for me to think about the map, maybe for another person it is not.” (P03 in the group interview) "I feel that a map (…) follows a list of problems. We move from the list to a graphical representation that allows relationships to be established between different health problems. It also allows the tutor to visualize and give feedback to the students (…). Therefore, I can see that CMs could be a very powerful medical education tool (…)." (P14 in focus group) “We could always use be the same CM for teaching and in the clinical setting. In this "unique CM", we would be able to use different magnifications on different contents, according to the focal question of the map, addressing different knowledge and complexity levels. It could even be a set of several CMs that are interconnected, forming a learning spiral method. (P10 in focus group)

CM, concept map; P, participant

**Table 3:** Quotations regarding the CMs as a tool to support management and treatment plan

Code	Illustrative examples
*CMs support management and treatment plan*	
Management Plan	"CMs are useful in complex patients or for presenting clinical cases (…), as they allow us to mentally visualize the patient and outline our actions (…)". (P02 in the group interview) "I have the perception that it is only now that I am able to have enough knowledge and experience to be able to use CMs (…), that is, to use them with all their potential to make a decision." (P15 in focus group)
Drug Interactions	"The construction of the individual CM allowed a better management of pharmacological treatment (drug interactions, side-effects).” (gallery walk exercise) "(…) I had never included drugs associated with diseases on the map (…) but it was useful. We are visualizing the diseases associated with the respective drugs. It is useful in polymedicated patients, to help identify possible adverse effects (…)." (P15 in focus group)

CM, concept map; P, participant

**Table 4:** Quotations regarding the CMs as a tool to promote collaborative learning

Code	Illustrative examples
*CMs promote collaborative learning*	
Collaborative Learning	“CMs were a collaborative learning promoting tool.” (post-it note from the wall of values) “The group task helped to improve clinical reasoning skills. By comparing the individual CMs, they were able to identify different perspectives and to see the links between different concepts.” (P02 in the group interview)
Knowledge Gaps	“The construction of the group CM promoted knowledge gaps identification.” (gallery walk exercise)
Group Brainstorming	“The construction of the group CM promoted group brainstorming.” (gallery walk exercise) “Many people in the group were initially concerned about the quality of their individual CMs. They felt that their maps were disorganized and that no one would be able to understand them. However, when it came time to share the maps with the group, we were much more confident and capable of making connections between concepts.” (P03 in the group interview)
Clinical Case Discussions	"I think that if, for example, I were asked as a resident to transmit the information of a patient to the rest of the medical team, it might be a useful tool (…)." (P03 in the group interview)

CM, concept map; P, participant

#### CMs facilitate clinical information integration

The students’ evaluation of CMs in the gallery walk exercise showed that CMs were effective tools for facilitating *identification*, *organization*, and *synthesis* of clinical information (Table 2). This potential to synthesize clinical information was also mentioned as an advantage for the future use of CMs. Despite students perceiving these points as beneficial, organizing concepts and selecting relevant information was difficult.

The visual representation of information, organized hierarchically, promoted a better *integration* of the patient’s clinical information, which can also promote a holistic approach that characterizes Family Medicine (mentioned in the gallery walk exercise and in some post-it notes). Several students and tutors highlighted this particularity of the *visual representation* as a way for tutors to guide students. Most of the students preferred the use of graphical diagrams:

- "CM allows us to establish clinical reasoning and the relationship between various diseases. It's something that a list, a text, could not do in any way." (Participant (P) 03 in the group interview)

However, a few students preferred text narratives over graphical representations. The research team approached these participants informally and realized that they considered this tool too schematic and valued more text narratives. These individual preferences and the feeling that creating the maps was time-consuming were the reasons mentioned by the students and collected by the research team in their field notes, which were confirmed in the interviews.

Students mentioned that CMs were particularly useful in the process of integrating and synthesizing clinical information:

- "Without CMs, it wouldn't be possible to see the relationship between the patient's pathologies so well, everything that contributes to a greater cardiovascular risk and let's not forget concepts and connections (…)." (P01 in the group interview)

In the difficult task of managing patients with multimorbidity, where multipathology and polypharmacy are present, CMs helped to better integrate information. CMs were considered simplifiers of complexity, making complex clinical cases more manageable. This point was also valued by the tutors who perceived the maps as being a powerful medical education tool. Several participants mentioned that CMs supported clinical reasoning. The visual nature of CMs made them particularly effective in facilitating decision-making, which is directly related to the following educational impact.

During the focus group session, some tutors reported that the CMs used in real clinical settings appeared to depict a different reality compared with their application in undergraduate education. P15 expressed the belief that there are distinct CMs tailored for teaching purposes and others designed in clinical practice, asserting that they represent "two very different things". P10 suggested that a single CM could potentially serve both purposes. This "unique CM" could accommodate varying levels of magnification depending on the specific topic, thereby addressing different levels of knowledge and complexity. Moreover, it could even consist of a series of interconnected CMs, forming a spiral learning approach. P13 agreed and commented that she had difficulty distinguishing CMs as pedagogical tools from CMs used in clinical practice, as she perceives them to be essentially the same.

#### CMs support management and treatment plan

Following the previous point, both in the gallery walk and in the post-it notes exercises, the students mentioned that CMs were helpful in planning the *patient care plan* (Table 3). The integration of clinical information, including data from the medical history and observation, allowed them to better understand the patient. This information could be used to create a comprehensive picture of the patient’s condition and move on to the decision-making step. Regarding pharmacological treatment, CMs were perceived as a useful tool to *detect drug interactions and side effects*. In addition, it was helpful in showing how different conditions and drugs influenced each other, visualizing how drugs could have unexpected adverse effects on conditions other than those for which the drug was prescribed:

- "With CMs I was able to see the patient as a whole (…) and I had the feeling that I was relating things much more easily. (…) The biggest advantage is connecting medications, pathologies, personal history and being able to see this on the map." (P07 in the group interview)

#### CMs promote collaborative learning

CMs were found to be a valuable tool for promoting *collaborative learning*, as highlighted by the students in the gallery walk and in the post-it notes exercises (Table 4). The students found the interactive nature of these activities to be engaging and effective in facilitating meaningful discussions between different perspectives:

- "In the group task, it was advantageous to compare the individual CMs. Sometimes I start with one concept, and my colleague may start with a different one. (…) I think it is important to discuss the links between the different pathologies and the different risk factors in a group, because it is a form of learning. (…)." (P05 in the group interview)

Students mentioned that they would like to have the opportunity to compare and discuss the group CMs in the whole group setting. Moreover, the construction of group CMs was also useful for identifying *knowledge gaps* in a dynamic environment for knowledge exchange, where students shared their understanding and perspectives and could identify areas where their knowledge was lacking or where inconsistencies existed. This group task, pre-prepared by the construction of individual CMs, encouraged *group brainstorming*, linking concepts, and exploring relationships, thus fostering the generation of novel ideas and approaches. This collaborative brainstorming could lead to the discussion of clinical problems, facilitate *clinical case discussions*, improve patient management, and enhance students’ problem-solving and clinical reasoning skills.

### Resources for implementing CMs

In this study we identified several key resources necessary for the effective implementation of CMs. Time was a significant factor, with no CM completed, as participants noted that there was "not enough time to perform the task" (mentioned in the gallery walk exercise). Group discussions were also highlighted as beneficial. Clear instructions were deemed essential, with participants referring to "strict CMs construction rules" as necessary for guidance (mentioned in the gallery walk exercise). The use of software emerged as a valuable tool. Lastly, feedback from tutors was identified as crucial for learning and improvement (more detailed information is in Additional Supporting Information 7).

## Discussion

We conducted a qualitative study in a Portuguese medical school to delineate how CMs can facilitate clinical reasoning in multimorbidity patients within undergraduate Family Medicine curricula, as perceived by 5th-year students and tutors. We also aimed to identify the resources required for CMs implementation. We found that CMs promoted the integration of clinical information, supported the development of management and treatment patient plan, and fostered collaborative learning. To optimize the implementation of CMs, we identified several key elements: providing clear instructions for map construction, using user-friendly software, allocating sufficient time for the task, encouraging group discussion of CMs, and incorporating tutor feedback throughout the map creation process.

Our study demonstrated that CMs facilitated the integration of clinical information by aiding in identification, organization, synthesis, and visualization. This finding aligns with previous research (Choudhari et al., 2021; Kumar et al., 2011). This integration of clinical information was particularly valuable in managing complex cases with multimorbidity: students and tutors appreciated the ability to synthesize and visualize relevant information in these complex cases. Furthermore, CMs supported the management and treatment planning process, particularly in understanding drug-drug and drug-pathology interactions in polypharmacy scenarios. This finding represents an original contribution to the literature in the context of clerkship undergraduate medical education, with prior references found only in nursing education (Hundial, 2020).

Considering the various components of clinical reasoning (Daniel et al., 2019), our study's findings illustrate how ‘CMs facilitate clinical information integration’, particularly in the *problem representation* component. The construction of a robust patient representation, encompassing biopsychosocial factors, is crucial for achieving a holistic understanding of a multimorbidity patient, and CMs serve as effective tools for simplifying this complexity. Furthermore, the theme ‘CMs support management and treatment plan’ corresponds well to the *management and treatment* component of clinical reasoning. The visual representation provided by the CMs helped students to pinpoint focal areas in the management plan and understand the potential impact of interventions on the patient’s overall health. It is important to highlight that the clinical vignette presented to students in the intervention sessions encompassed all the diagnoses. Consequently, the exercise was not designed to explore components such as information gathering, hypothesis generation, differential diagnosis, formulation of leading or working diagnoses, or diagnostic justification.

The potential of using a single and comprehensive CM (“unique CM”) as a visual roadmap for navigating clinical cases, representing health problems, and management and treatment was also referred. The maps could be tailored to specific content areas based on the specific learning objectives at a given time, throughout the various learning phases. This approach aligns with the potential utility of CMs in bridging the persistent gap between basic sciences and clinical practice for clerkship students (Peñuela-Epalza & Hoz, 2019). All these aspects can be beneficial for tutors as a means of monitoring students' learning progression, as noted in our study and by Bixler et al. (2015), Choudhari et al. (2021), Choudry et al. (2019), Silva Ezequiel et al. (2019), and others. Radwan et al. (2019) concluded that there was a significant correlation between 6th-year medical students' ability to construct CMs and their clinical reasoning skills, suggesting the potential use of CMs as an assessment tool for evaluating clinical reasoning skills.

Regarding collaborative learning, several studies have highlighted the promotion of collaborative learning by CMs and considered them a valuable tool for educators (Bixler et al., 2015; Peñuela-Epalza & Hoz, 2019; Saeidifard et al., 2014; Silva Ezequiel et al., 2019; Torre et al., 2017a). In our study, the group activity was highly valued by the students as it fostered a space for integrating diverse perspectives and strengthening clinical reasoning skills through the use of CMs. The research team observed a high level of student motivation during this task, with most students actively engaged in the activity. Additionally, the tutors who participated in the focus group, from two medical schools with different curricula, shared a consensus on the usefulness of CMs. While these identified educational impacts appear to be related to the use of CMs, we must acknowledge that the way we implemented the intervention also might have played a significant role. A control group would be useful for drawing more definitive conclusions.

Concerning the resources required to implement CMs, we would like to highlight several key points. First, clearer guidelines for CM construction are crucial. The 10-min lecture on how to build CMs proved insufficient for first-time users. Detailed instructions and opportunities for hands-on practice are essential to facilitate effective CM creation. Second, sufficient time allocation for the task is critical. Students required more time to complete the maps. In future sessions, we recommend allocating at least 40 min for individual CM construction and another 40 min for group CM construction. Third, incorporating tutor feedback throughout the map creation process is vital. This aspect was highly valued in our study because it may amplify the benefits of using CMs. This aligns with findings described by other groups (Ben-Haddour et al., 2022; Kumar et al., 2011). Tutor feedback can guide students toward creating more accurate and comprehensive CMs, ultimately enhancing their learning experience. Additionally, it is essential to extend the intervention over a longer period, allowing students to become more familiar with the tool and improve their application, with more opportunities to receive the feedback.

The resulting CMs were generally simpler than anticipated, likely due to the limited time allocated to the task, as well as other factors such as lack of prior training and insufficient feedback from tutors. This observation suggests that effective CM use is time intensive. A small number of students also stated that CM construction was time-consuming. This finding is not surprising and has been mentioned in other studies (Baliga et al., 2021; Torre et al., 2017a). However, Veronese et al. (2013), in their randomized study incorporating CMs into problem-based learning tutorials, noted that initial resistance to CMs often fades as students recognize their benefits.

The lessons learned from this study for our group, with a view to subsequent large-scale implementation, involve a thorough review of the educational intervention session. It will be important to evaluate other components of clinical reasoning, such as information gathering, hypothesis generation, differential diagnosis, formulation of leading or working diagnoses, and diagnostic justification. This will require adjusting the time for session tasks, such as allocating more time for CMs construction after a few training sessions on map-building techniques and using software instead of requesting paper-based maps. Providing feedback to students as they build CMs necessitates implementation across multiple sessions. Having a control group would also be relevant. For other groups, we emphasize that this tool should be adjusted to the context and intended objectives. CMs are most useful in a reflective context with students in a controlled training environment without the pressure of a real patient. Like all tools in medical education, their selection should be thoughtful, and their use should depend on the objectives.

Limitations included a low student participation rate, which impacts dependability and transferability. The insufficient time allocated for students to create CMs, which probably limited their ability to fully integrate the complexities of multimorbidity, potentially reducing the effectiveness of CMs in developing clinical reasoning skills. Adaptations from planned focus groups to semi-structured group interviews due to low turnout may have affected the depth of the data. Although these interviews provided valuable insights, the shift from interactive focus groups to more structured interviews might have altered participant interactions. Additionally, the research team’s prior experience with CMs, though managed through reflexivity, could have influenced interpretations. Scoring the CMs could have provided more quantifiable results, but this point is still being debated among researchers, and a standardized method is lacking (Brondfield et al., 2021; Torre et al., 2017b; West et al., 2002).

## Conclusions

CMs are pedagogical tools that promote the interconnection of concepts, effectively facilitating clinical information integration and supporting management and treatment plans. This visual representation helps students better understand multimorbidity patients in the context of undergraduate medical education, promoting components of clinical reasoning such as problem representation and patient management and treatment.

## Supplementary Information

Below is the link to the electronic supplementary material.

Supplementary file1 (PDF 19 KB)

Supplementary file2 (PDF 91 KB)

Supplementary file3 (PDF 408 KB)

Supplementary file4 (PDF 107 KB)

Supplementary file5 (PDF 153 KB)

Supplementary file6 (PDF 116 KB)

Supplementary file7 (PDF 99 KB)

## Footnotes

1 An Erasmus student is someone participating in the Erasmus program, an initiative by the European Union that allows students to study or train in another European country for a period ranging from a few months to a full academic year.
