# Supplementary material for: Promoting clinical reasoning in undergraduate Family Medicine curricula through concept mapping: a qualitative approach
Source: Adv Health Sci Educ Theory Pract. 2024 Jun 24;30(2):383–400. doi: 10.1007/s10459-024-10353-z (PMC11965178; doi:10.1007/s10459-024-10353-z)
Supplement: Supplementary file 1 — Supplementary file1 (PDF 19 KB) [file 10459_2024_10353_MOESM1_ESM.pdf]

Additional Supporting Information 1

Article Title Promoting clinical reasoning in undergraduate Family Medicine curricula through concept mapping: a qualitative approach.
Journal Name Advances in Health Science Education
Authors Marta Fonseca[1,2], Pedro Marvão[2], Patrícia Rosado-Pinto[2], António Rendas[2], Bruno Heleno[1,2]
Affiliations [1] Comprehensive Health Research Centre, Lisbon, Portugal; [2] NOVA Medical School, Lisbon, Portugal
Corresponding author Marta Fonseca, marta.fonseca@nms.unl.pt

Clinical vignette

Male patient, 54 years old, born in Bangladesh, residing in Portugal for 14 years.
He has some difficulty understanding and speaking Portuguese.
He is married and has 5 children (the 2 oldest live in Bangladesh).
He is a street vendor.

He denies smoking, alcohol, or drug use.

He reports being diagnosed with **hypertension** at the age of 40. He was prescribed **amlodipine** 5mg twice daily but admits he is taking it only occasionally because he feels well. He also states that he has not been monitoring his blood pressure since the diagnosis.

At the age of 42, he was hospitalized due to an upper gastrointestinal hemorrhage. A **gastric ulcer** was diagnosed during the hospitalization. His blood pressure was controlled during the hospitalization only with dietary modifications.

Family history:
The patient's father died of an acute myocardial infarction.
The patient denies any other family history of significant medical conditions.

**S**	The patient presents for a follow-up appointment with the general practitioner to monitor his blood pressure and discuss recent lab test results. He does not regularly monitor his blood pressure at home.<br>He reports **left knee pain** for the past week. He has been taking **diclofenac** 50 mg, three times daily, for one week for pain control.
**O**	Physical Examination:<br>Weight 82 kg, height 1.67 m, BMI 29.4 kg/m², blood pressure 160/90 mmHg, heart rate 82 bpm, normal auscultation of the heart and lungs, mild bilateral malleolar edema.<br><br>Laboratory Results:<br>Glucose: 290 mg/dl, Total cholesterol: 283 mg/dl, HDL cholesterol: 29 mg/dl, Triglycerides: 178 mg/dl, Urinalysis: 2+ glycosuria (1000 mg/dl).<br>Fasting glucose (performed about 1 month ago): 257 mg/dl.<br><br>ECG: no abnormalities.<br>Knee X-ray: bilateral gonarthrosis, more severe on the left.
**A**	Hypertension (HTA) diagnosed 14 years ago, currently uncontrolled<br>Type 2 diabetes mellitus<br>Dyslipidemia<br>Overweight (BMI 29.4 kg/m²)<br>Osteoarthritis of the knees<br>Poor medication adherence<br>Language barrier
**P**	Medications: initiate metformin, initiate enalapril, paracetamol + tramadol for pain control.<br>Order laboratory tests, including HbA1C.<br>Schedule follow-up appointment to monitor diabetes control and overall health.
