# Supplementary material for: Promoting clinical reasoning in undergraduate Family Medicine curricula through concept mapping: a qualitative approach
Source: Adv Health Sci Educ Theory Pract. 2024 Jun 24;30(2):383–400. doi: 10.1007/s10459-024-10353-z (PMC11965178; doi:10.1007/s10459-024-10353-z)
Supplement: Supplementary file 2 — Supplementary file2 (PDF 91 KB) [file 10459_2024_10353_MOESM2_ESM.pdf]

Additional Supporting Information 2

Article Title Promoting clinical reasoning in undergraduate Family Medicine curricula through concept mapping: a qualitative approach.

Journal Name Advances in Health Science Education

Authors Marta Fonseca[1,2], Pedro Marvão[2], Patrícia Rosado-Pinto[2], António Rendas[2], Bruno Heleno[1,2]

Affiliations [1] Comprehensive Health Research Centre, Lisbon, Portugal; [2] NOVA Medical School, Lisbon, Portugal

Corresponding author Marta Fonseca, marta.fonseca@nms.unl.pt

Group interviews questions (students):

1. Overall impressions of the intervention session.
2. Positive aspects of the experience of using concept maps, and their contribution to learning, clinical information integration, and reflection on the clinical vignette.
3. What difficulties were encountered in constructing the maps? Is this an intuitive tool?
4. How do you see the use of this tool in the future, both as a student and later in clinical practice? How could it be useful? What aspects should be considered?

Focus Group Agenda (tutors):

1. Introduction of participants. Verification of informed consent submissions. Start recording.
2. Brief introduction with a summary of the research project and the methodology of the intervention session.
3. Open discussion in the group / reflection:
    a. General comments
    b. Comments on the project
    c. Comments on the use of concept maps in the teaching of Family Medicine, as a tool to facilitate the integration of clinical information in complex patients
4. Personal experiences with the use of concept maps.
    a. Advantages
    b. Disadvantages
5. Final comments and conclusion. Thanks to the participants. Closing of the session.
