# Supplementary material for: Promoting clinical reasoning in undergraduate Family Medicine curricula through concept mapping: a qualitative approach
Source: Adv Health Sci Educ Theory Pract. 2024 Jun 24;30(2):383–400. doi: 10.1007/s10459-024-10353-z (PMC11965178; doi:10.1007/s10459-024-10353-z)
Supplement: Supplementary file 4 — Supplementary file4 (PDF 107 KB) [file 10459_2024_10353_MOESM4_ESM.pdf]

Additional Supporting Information 4

Article Title Promoting clinical reasoning in undergraduate Family Medicine curricula through concept mapping: a qualitative approach.

Journal Name Advances in Health Science Education

Authors Marta Fonseca[1,2], Pedro Marvão[2], Patrícia Rosado-Pinto[2], António Rendas[2], Bruno Heleno[1,2]

Affiliations [1] Comprehensive Health Research Centre, Lisbon, Portugal; [2] NOVA Medical School, Lisbon, Portugal

Corresponding author Marta Fonseca, marta.fonseca@nms.unl.pt

Results from the gallery walk exercise:

**Station 1**	**How was the construction of the individual CM useful in integrating the clinical information from the vignette?**	
	Identification, organization, and synthesis of the patient's clinical information	4
	Integration of the clinical information / holistic approach	3
	Better management of pharmacological treatment (drug interactions, side-effects)	2
	Hierarchical organization of the patient problems	1
	Visual representation of the clinical information	1
	Time consuming activity	1
**Station 2**	**How was the group discussion and construction of the final CM useful?**	
	Knowledge gaps identification	2
	Promotes improving the group CM with the integration of different perspectives	2
	Group discussion promotes collaborative learning	1
	Promotes clinical reasoning	1
	Promotes group brainstorming	1
**Station 3**	**What were the main difficulties experienced when constructing the CM?**	
	Conceptual organization	4
	Selection of the relevant clinical information	3
	Not enough time to perform the task	2

Additional Supporting Information 4

	Strict CMs construction rules	1
Station 4	**How would you like to use CMs in the future?**	
	Synthesis of the patient's clinical information with multimorbidity	3
	Visual integration of the clinical information, promoting a holistic approach	3
	Clinical case discussions	2
	Learning method	1

Abbreviations: CM, concept map.
