# Supplementary material for: Promoting clinical reasoning in undergraduate Family Medicine curricula through concept mapping: a qualitative approach
Source: Adv Health Sci Educ Theory Pract. 2024 Jun 24;30(2):383–400. doi: 10.1007/s10459-024-10353-z (PMC11965178; doi:10.1007/s10459-024-10353-z)

Additional Supporting Information 5

Article Title Promoting clinical reasoning in undergraduate Family Medicine curricula through concept mapping: a qualitative approach.

Journal Name Advances in Health Science Education

Authors Marta Fonseca[1,2], Pedro Marvão[2], Patrícia Rosado-Pinto[2], António Rendas[2], Bruno Heleno[1,2]

Affiliations [1] Comprehensive Health Research Centre, Lisbon, Portugal; [2] NOVA Medical School, Lisbon, Portugal

Corresponding author Marta Fonseca, marta.fonseca@nms.unl.pt

Wall of values with the student's post-it notes written at the end of the intervention session, with what they most valued from the session.

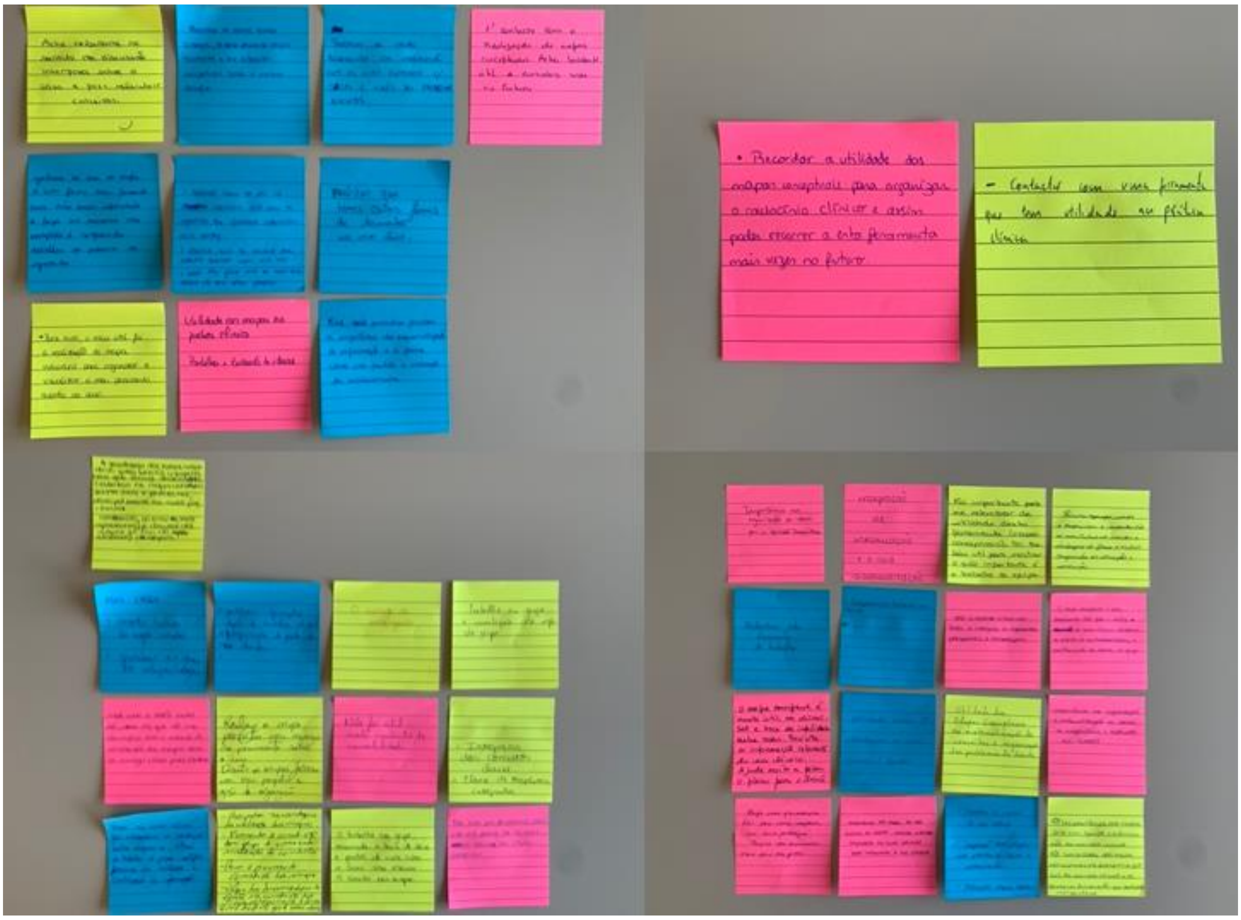

Additional Supporting Information 5

Results of the students post-it notes from the wall of values:

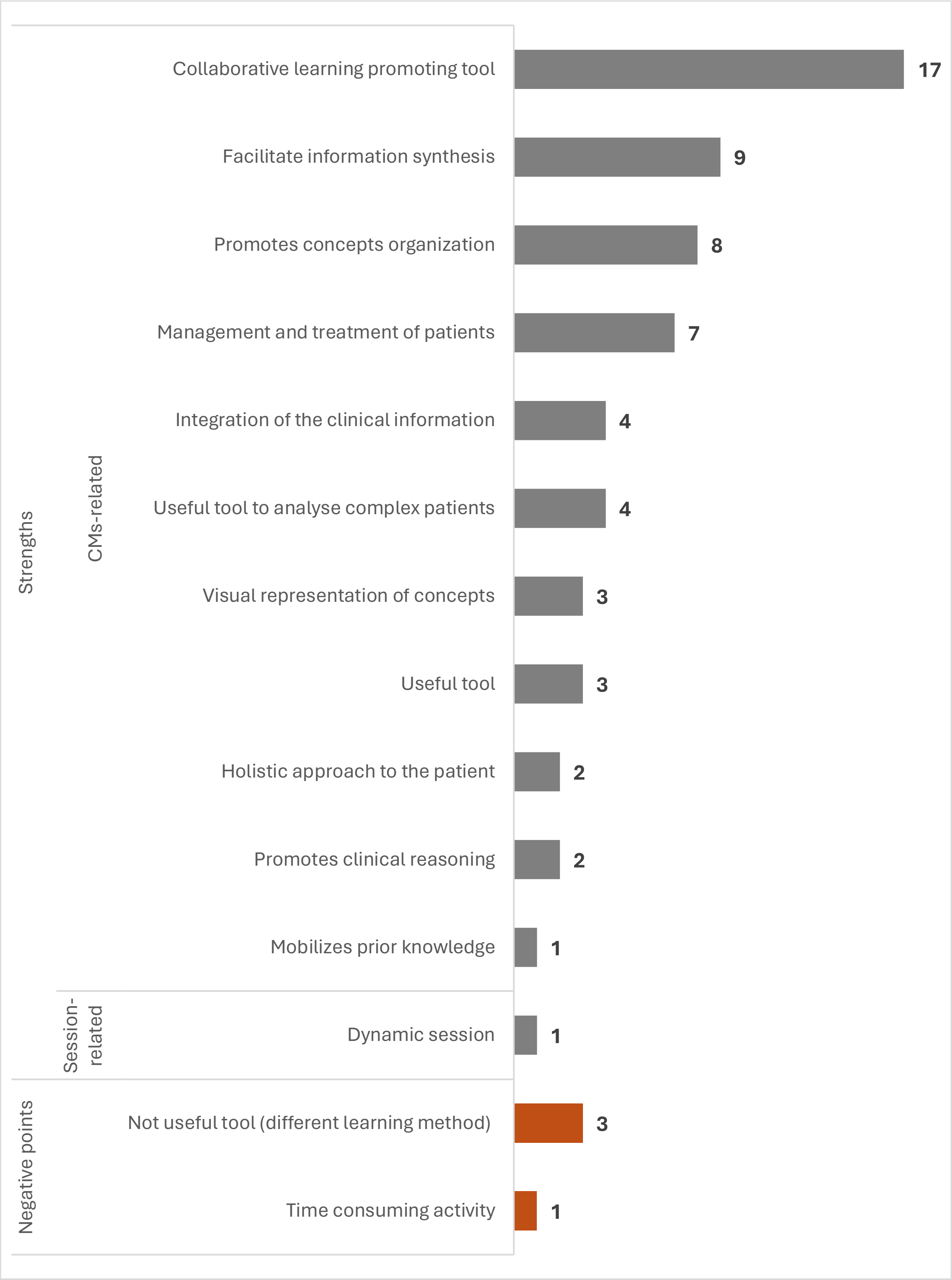

Supplement: Supplementary file 5 — Supplementary file5 (PDF 153 KB) [file 10459_2024_10353_MOESM5_ESM.pdf]
