# Supplementary material for: Promoting clinical reasoning in undergraduate Family Medicine curricula through concept mapping: a qualitative approach
Source: Adv Health Sci Educ Theory Pract. 2024 Jun 24;30(2):383–400. doi: 10.1007/s10459-024-10353-z (PMC11965178; doi:10.1007/s10459-024-10353-z)
Supplement: Supplementary file 6 — Supplementary file6 (PDF 116 KB) [file 10459_2024_10353_MOESM6_ESM.pdf]

Additional Supporting Information 6

Article Title Promoting clinical reasoning in undergraduate Family Medicine curricula through concept mapping: a qualitative approach.

Journal Name Advances in Health Science Education

Authors Marta Fonseca[1,2], Pedro Marvão[2], Patrícia Rosado-Pinto[2], António Rendas[2], Bruno Heleno[1,2]

Affiliations [1] Comprehensive Health Research Centre, Lisbon, Portugal; [2] NOVA Medical School, Lisbon, Portugal

Corresponding author Marta Fonseca, marta.fonseca@nms.unl.pt

Quotations from the group interviews and focus group:

**Code**	**Description**	**Illustrative examples**
CMs facilitate clinical information integration		
Identification	Identification of relevant information from the clinical case	"(...) the individual map was more for me to familiarize myself with the patient." (P01 in the group interview)<br><br>"The idea of the map is to highlight the different health problems, (...) experienced by me as the physician (...)." (P10 in focus group)<br><br>"(...) the objective is to identify entities, not only issues that are related to the physician's agenda, but that are related to the patient's agenda, which identify priorities for manage a plan later." (P13 in focus group)
Organization	Organization of relevant concepts of clinical information	"CMs allow for better organization and visualization of the patient." (P02 in the group interview)<br><br>"(...) organizing ideas is easier with CMs. I make diagrams or tables too." (P04 in the group interview)<br><br>"(...) and I think the final map was even more organized, because everyone made their contribution and also made an effort to make it as organized as possible (...)." (P07 in the group interview in relation to group CMs)<br><br>"(...) in other words, it is as if this CM managed, in a very clear and hierarchical way, to almost decompose some causes that then determine some mechanisms of the disease and the pathogenesis that then leads to these manifestations." (P14 in focus group)
Synthesis	Synthesis of a patient's clinical information	"(...) when I have a clinical case to present, I will often create a CM to summarize the patient's information." (P03 in the group interview)
Integration	Connects concepts, ideas, and relationships of the clinical information	"CM allows the integration of all patient factors." (P02 in the group interview)<br><br>"Without CMs, it wouldn't be possible to see the relationship between the patient's pathologies so well, everything that contributes to a greater cardiovascular risk and, and let's not forget concepts and connections (...)." (P01 in the group interview)<br><br>"CM allows establishing relationships between various pathologies and polypharmacy in the context of multimorbidity." (P03 in the group interview)<br><br>"I think the advantage of CMs is to integrate all the information, (...) in a short space of time, and we are able to have a global view of the patient." (P04 in the group interview)<br><br>"With CMs I was able to see the patient as a whole (…) and I had the feeling that I was relating things much more easily. For example: a patient who has anemia because he has an ulcer due to taking anti-inflammatories

		for several years. The biggest advantage is connecting medications, pathologies, personal history and being able to see this on the map." (P07 in the group interview)<br><br>"I can only make a good problem map to support the therapeutic plan if I can make this concept map. If I can't make the concept map, this background issue, I can't resolve the foreground issues." "(...) this map of concepts can, in a very clear and hierarchical way, identify some causes that determine some mechanisms of the disease and the pathogenesis of the manifestations." (P14 in focus group)
Visual Representation	Schematic representation of concepts and their relationships	"CMs ultimately allow for better organization and visualization of the patient." (P02 in the group interview)<br><br>"CM allows us to establish clinical reasoning and the relationship between various diseases. It's something that a list, a text, could not do in any way." (P03 in the group interview)<br><br>"(...) visually it is much easier." (P05 in the group interview)<br><br>"It is an individual matter, that is, there are people who identify very much with CMs, I happen to be one of them, but there are others who identify much more with text. (...) while it is very easy for me to think about the map, maybe for another person it is not." (P03 in the group interview)<br><br>"I feel that a map (...) follows a list of problems. We move from the list to a graphical representation that allows relationships to be established between different health problems. It also allows the tutor to visualize and give feedback to the students (...). Therefore, I can see that CMs could be a very powerful medical education tool (...)." (P14 in focus group)<br><br>"We could always use be the same CM for teaching and in the clinical setting. In this "unique CM", we would be able to use different magnifications on different contents, according to the focal question of the map, addressing different knowledge and complexity levels. It could even be a set of several CMs that are interconnected, forming a learning spiral method. (P10 in focus group)
CMs support management and treatment plan		
Management Plan	Management of care and therapeutic plan	"CMs are useful in complex patients or for presenting clinical cases (...), as they allow us to mentally visualize the patient and outline our actions (...)". (P02 in the group interview)<br><br>"I have the perception that it is only now that I am able to have enough knowledge and experience to be able to use CMs (...), that is, to use them with all their potential to make a decision." (P15 in focus group)
Drug Interactions	Side effects, adverse reactions, and interactions with other diseases	"(...) CMs make it easier to see if there are drug interactions and possible adverse effects that may be contributing to the worsening of certain pathologies." (P03 in the group interview)<br><br>"With CMs I was able to see the patient as a whole (…) and I had the feeling that I was relating things much more easily. For example: a patient who has anemia because he has an ulcer due to taking anti-inflammatories for several years. The biggest advantage is connecting medications, pathologies, personal history and being able to see this on the map." (P07 in the group interview)<br><br>"(...) I had never included drugs associated with diseases on the map (...) but it was useful. We are visualizing the diseases associated with the respective drugs. It is useful in polymedicated patients, to help identify possible adverse effects (...)." (P15 in focus group)
CMs promote collaborative learning		
Collaborative Learning	Students learn working together in small groups	"The group task helped to improve clinical reasoning skills. By comparing the individual CMs, they were able to identify different perspectives and to see the links between different concepts." (P02 in the group interview)<br><br>"In the group task, it was advantageous to compare the individual CMs. Sometimes I start with one concept, and my colleague may start with a different one. (...) I think it is important to discuss the links between the different pathologies and the different risk factors in a group, because it is a form of learning. I think we always have something to learn from others,

Additional Supporting Information 6

		and it always broadens our horizons. That's why I think the CM that was developed in a group is more complete (...)."(P05 in the group interview)
Knowledge Gaps	Individual lacks understanding of concepts identified in the group task	“This helped to identify gaps in knowledge and to develop a more complete understanding of the topic.” (P02 in the group interview)
Group Brainstorming	Ideas generated within a group through an unstructured way	“Many people in the group were initially concerned about the quality of their individual CMs. They felt that their maps were disorganized and that no one would be able to understand them. However, when it came time to share the maps with the group, we were much more confident and capable of making connections between concepts.” (P03 in the group interview)
Clinical Case Discussions	Group sharing of information and perspectives to develop a plan of care	"I think that if, for example, I were asked as a resident to transmit the information of a patient to the rest of the medical team, it might be a useful tool (...)." (P03 in the group interview)

Abbreviations: CM, concept map; P, participant.
