# Supplementary material for: Promoting clinical reasoning in undergraduate Family Medicine curricula through concept mapping: a qualitative approach
Source: Adv Health Sci Educ Theory Pract. 2024 Jun 24;30(2):383–400. doi: 10.1007/s10459-024-10353-z (PMC11965178; doi:10.1007/s10459-024-10353-z)
Supplement: Supplementary file 7 — Supplementary file7 (PDF 99 KB) [file 10459_2024_10353_MOESM7_ESM.pdf]

## Additional Supporting Information 7

**Article Title** Promoting clinical reasoning in undergraduate Family Medicine curricula through concept mapping: a qualitative approach.

**Journal Name** Advances in Health Science Education

**Authors** Marta Fonseca[1,2], Pedro Marvão[2], Patrícia Rosado-Pinto[2], António Rendas[2], Bruno Heleno[1,2]

**Affiliations** [1] Comprehensive Health Research Centre, Lisbon, Portugal; [2] NOVA Medical School, Lisbon, Portugal

**Corresponding author** Marta Fonseca, marta.fonseca@nms.unl.pt

Quotations related with **resources** for implementing CMs:

| **Code** | **Description** | **Illustrative examples** |
|---|---|---|
| Resources | Time | “Not enough time to perform the task.” (gallery walk exercise) |
| | Group discussions | "In my opinion, it would be beneficial to conclude the session with each group presenting their CMs and engaging in a peer-to-peer discussion of the maps." (P03 in the group interview) |
| | Instructions | “Strict CMs construction rules.” (gallery walk exercise) |
| | Software | “Creating concept maps on paper can be quite restrictive, whereas using a software program on a computer is a much simpler process. It might take longer initially, but it becomes significantly easier to organize and add more information as you progress." (P05 in the group interview) |
| | Feedback from tutors | “Upon completing an exercise and receiving feedback such as, 'Okay, how did you solve it?', we can always learn something new." (P07 in the group interview) |

Abbreviations: CM, concept map; P, participant.
